# Copy number variation of whole genome sequence in 1519 patients with Alzheimer’s disease in China

**DOI:** 10.1002/alz.087393

**Published:** 2025-01-03

**Authors:** Lu Shen, Qijie Yang, Xuewen Xiao, Tianyan Xu, Yuan Zhu, Bin Jiao

**Affiliations:** ^1^ Xiangya Hospital, Central South University, Changsha, Hunan China

## Abstract

**Background:**

Single nucleotide polymorphism (SNP)‐based genetic studies have identified many risk genes for Alzheimer’s disease (AD), but only explain part of the heritability. Structural variation (SVs) may account for some of this otherwise unexplained heritability. In this study, we sequenced 1,519 AD patients and 2,010 controls using 30X whole‐genome sequencing (WGS).

**Method:**

Using six complementary CNV callers, we generated a high‐confidence CNV set. We reviewed rare variants within the reported 117 causal genes for dementia in our cohort. We also assessed the linkage disequilibrium (LD) between single nucleotide variants (SNVs) and copy number variants (CNVs).

**Result:**

We identified 66990 deletions and 10906 duplications. On dementia genes, we observed the a total of 28 AD patients carried with rare CNVs in ***APP*** (duplication, 1 EOAD, Known pathogenicity), ***OPTN*** (deletion, 6 AD and 2 control), ***PRKAR1B*** (duplication, 1 EOAD), ***VPS13A*** (deletion, 1 EOAD), and et al. LD Analysis revealed that a 5.2 Kb deletion in *NBEAL1* intron highly LD (R2 = 0.95) with rs139643391 (chr2:202878716) and a 291bp deletion in *TMEM106B* highly LD (R2 = 0.97) with rs5011436 (chr7: 12229132).

**Conclusion:**

Overall, our findings suggest that the occurrence of *APP* locus duplication appears to be less common in the Chinese population. Other rare CNV variants in dementia genes may have potentially pathogenic and further validation of their pathogenicity is needed.

